# OCLN as a novel biomarker for prognosis and immune infiltrates in kidney renal clear cell carcinoma: an integrative computational and experimental characterization

**DOI:** 10.3389/fimmu.2023.1224904

**Published:** 2023-09-22

**Authors:** Zongming Jia, Ying Kong, Chengyu Wang, Zhenyu Fu, Zhen Tian, Yizhang Sun, Yuxin Lin, Yuhua Huang

**Affiliations:** ^1^ Department of Urology, The First Affiliated Hospital of Soochow University, Suzhou, China; ^2^ Center for Systems Biology, Department of Bioinformatics, School of Biology and Basic Medical Sciences, Soochow University, Suzhou, China

**Keywords:** OCLN, renal clear cell carcinoma, prognostic biomarker, immune infiltration, translational informatics

## Abstract

**Background:**

Occludin (OCLN) is an important tight junction protein and has been reported to be abnormally expressed in the development of malignant tumors. However, its biomarker and carcinogenic roles in kidney renal clear cell carcinoma (KIRC) are less investigated.

**Methods:**

The Cancer Genome Atlas database and Human Protein Atlas database were used to analyze the expression of OCLN in KIRC. UALCAN database and methylation-specific PCR assay were used to evaluate the methylation level of OCLN in KIRC. Univariate and multivariate Cox regression analyses were performed to model the prognostic significance of OCLN in KIRC patient cohorts. The correlation between OCLN expression and the immune cell infiltration, immune-related function and immune checkpoints were explored. Finally, EdU, scratch assay and transwell experiments were conducted to validate the role of OCLN in KIRC development.

**Results:**

The expression of OCLN was significantly downregulated in KIRC, compared with normal renal tissues (p<0.001). Patients with low OCLN expression showed a worse prognosis and poorer clinicopathological characteristics. Functional enrichment analysis revealed that OCLN was mainly involved in biological processes such as immune response, immunoglobulin complex circulating and cytokine and chemokine receptor to mediate KIRC development. Immune-related analysis indicated that OCLN could potentially serve as a candidate target for KIRC immunotherapy. OCLN overexpression inhibited proliferation, migration and invasion of KIRC cells *in vitro*.

**Conclusion:**

OCLN was validated as a candidate prognostic biomarker and therapeutic target of KIRC based both on computational and experimental approaches. More *in vivo* experiments will be conducted to decode its molecular mechanism in KIRC carcinogenesis in the future work.

## Introduction

The incidence of renal cell carcinoma (RCC) is rising gradually and it accounts for approximately 3% of all cancer types (1). The cause of RCC has not been well explored, but it may be a consequence of disorder both at genetic level and non-genetic factors such as smoking, alcohol consumption and obesity (2). As the most common subtype of RCC, kidney clear cell renal cell carcinoma (KIRC) is characterized with poor prognosis, severe clinical symptoms and potential treatment vulnerabilities (3). Currently, surgical resection has been proved to be the most effective way for KIRC treatment. However, the therapeutic effect and late recurrence are far from expected. Patients with advanced KIRC are mainly treated with systemic drug therapy, supplemented by palliative surgery or radiotherapy for primary or metastatic tumors (4, 5). Accumulating evidence indicated that the progression and metastasis of many cancers are relevant to T cell dysfunction and immune checkpoint-related immunosuppression (6–8). Several immune checkpoint inhibitors (ICI) have already been applied (9–11) to clinical use. These checkpoint inhibitors could block the binding of PD-1 to PD-L1, PD-L2, or CTLA-4 to CD80/CD86, alleviating suppression of the immune response, including the tumor immune response. Although the application of ICIs in advanced KIRC has been shown to improve patient outcomes (12), sensitive and specific biomarkers are still limited for predicting the effect of immunotherapy.

Tight junctions are intercellular protein complexes that connect the cytoskeletons of neighboring cells, thereby maintaining tissue homeostasis and integrity. Recent findings revealed the role of tight junction proteins during KIRC tumorigenesis. For example, Owari et al. demonstrated that CLDN4 phosphorylation induced nuclear translocation of YAP with CLDN4, thereby increasing the aggressiveness and metastatic capacity of renal cancer cells (13). Claudin-7 is the main component of tight junctions in epithelial cells, and Li et al. found that the overexpression of CLDN7 inhibited the proliferation, migration and invasion ability of KIRC cells *in vitro* and *in vivo (* 14). The OCLN gene encoded an integral membrane protein that is required for cytokine-induced regulation of the tight junction paracellular permeability barrier. In urological malignant tumors, OCLN is highly expressed in bladder urothelial carcinoma. Knockdown of OCLN is associated with proliferation inhibition and apoptosis promotion in bladder cancer cells *in vitro* and *in vivo (* 15). The tumor growth-promoting and metastatic effects of OCLN have also been demonstrated in prostate cancer, and the presence of lymphatic dissemination is also observed in the metastatic process (16). OCLN S490 phosphorylation regulates VEGF to induce endothelial proliferation and angiogenesis (17). However, the role of OCLN in KIRC has not been well determined yet.

In this study, an integrative computational and experimental analysis was performed to investigate the role of OCLN in KIRC progression. After verification of the Cancer Genome Atlas (TCGA) cohort and pathological sample through histological staining, OCLN was found to be one of the potential biomarkers for diagnosis and prognosis of KIRC. Immune-related analysis indicated that OCLN could serve as a potential target for immunotherapy in KIRC. The *in vitro* experiments of EdU, scratch assay and transwell experiments validated the computational findings and added the evidence of OCLN as a novel biomarker for KIRC management.

## Materials and methods

### Ethical approval

As shown in **Table S1**, a total of 21 cancer tissue and adjacent normal tissues were surgically collected from KIRC patients at The First Affiliated Hospital of Soochow University from January 2023 to April 2023. The samples were postoperatively pathologically confirmed. None of the patients had anti-tumor therapy prior to operation. This study was approved by the Ethics Committee of The First Affiliated Hospital of Soochow University with the number of 2023-181. All patients signed written informed consent.

### Gene expression and survival analysis

The expression of OCLN between tumor and adjacent normal tissues was analyzed by TIMER2 (18) (http://timer.cistrome.org/). The mRNA sequencing data including 542 KIRC specimens and 72 normal renal tissue specimens and the corresponding clinical information were downloaded from TCGA database on Mar, 23^rd^, 2023 (https://portal.gdc.cancer.gov/repository). As illustrated in **Table 1**, a total of 533 KIRC patients with full clinical information were included in this study. The R program (v4.2.2) was used to analyze the expression of OCLN between KIRC and normal kidney tissues and paired samples in TCGA-KIRC datasets, and the “ggpubr” and “ggplot2” R package were used for visualization. The “survival” R package was used for statistical analysis of survival data. The protein level and subcellular localization of OCLN was assessed by the Human Protein Atlas (HPA) database (19) (https://www.proteinatlas.org/).

**Table 1:** The clinical characteristics of TCGA-KIRC patients included in this study.

Characteristics	Freq(%)
Gender	No.(%)
Female	188 (35.3)
Male	345 (64.7)
Age	
<=65	349 (65.5)
>65	184 (34.5)
Overall survival status	
Alive	358 (67.2)
Dead	175 (32.8)
Histologic grade	
G1	14 (2.6)
G2	229 (43.0)
G3	206 (38.6)
G4	76 (14.3)
Gx	8 (1.5)
Pathologic stage	
Stage I	267 (50.1)
Stage II	57 (10.7)
Stage III	123 (23.1)
Stage IV	83 (15.6)
unknown	3 (0.6)
T	
T1	273 (51.2)
T2	69 (12.9)
T3	180 (33.8)
T4	11 (2.1)
N	
N0	240 (45.0)
N1	16 (3.0)
Nx	277 (52.0)
M	
M0	422 (79.2)
M1	79 (14.8)
Mx	32 (6.0)

### Clinical correlation analysis

The clinical data of TCGA-KIRC were used to analyze the correlation between the OCLN expression and the clinical characteristics of KIRC patients, including age, gender, tumor grade, clinical stage and TNM stage.

### Promoter methylation analysis

The OCLN promoter methylation level between KIRC and normal renal tissues was analyzed in the UALCAN database (20) (http://ualcan.path.uab.edu/index.html). Moreover, a stratified analysis based on patients’ age, gender, tumor grade, clinical stage, and N stage was performed.

### Functional enrichment analysis

The functional enrichment analysis of differentially expressed genes (DEGs) was performed at the gene ontology (21) (GO) and Kyoto Encyclopedia of Genes and Genomes (22) (KEGG) level using “ClusterProfiler” package (23) and “enrichplot” package in R. Gene set enrichment analysis (GSEA) (24) was used to explore the underlying molecular mechanisms. The terms with p<0.05 were considered to be statistically significant. The STRING platform (25) (v12.0) was used to screen genes potentially interacted with OCLN (https://cn.string-db.org/).

### Independent prognostic analysis and nomogram model construction

Univariate and multivariate Cox regression analysis were conducted using “survival” R package to evaluate the association between OCLN expression and KIRC prognosis. In addition, a nomogram was constructed by the “rms” R package (26) and “regplot” R package was applied to predict 1, 3 and 5 years overall survival (OS) in KIRC patients.

### Immune analysis

“CIBERSORT” package (27) was used to calculate the abundance across 22 immune cell subgroups in all samples. Samples with p<0.05 were chosen for further analysis to compare the differences in infiltration of various immune cells between the high and low OCLN expression groups. Kaplan-Meier plotter (http://kmplot.com/) databases were used to analyze the relationship between the expression of OCLN and OS in KIRC patients in different immune cell subsets. “ESTIMATE” was used to calculate the stromal score and immune score for KIRC. The relationship between stromal/immune score and OCLN expression was analyzed, the median value was set as the dividing line of high and low score. ssGSEA analysis was performed using the “GSEABase” and “GSVA” R packages to obtain scores for immune-related functions, and the scores were calibrated to compare differences in immune-related functions between high and low OCLN expression groups. The correlation between the OCLN expression and the expression level of immune checkpoint‐related genes was further analyzed.

### Western blot

Proteins were extracted from KIRC and adjacent normal renal tissues by RIPA lysate (Beyotime, China) supplemented with proteinase inhibitor. Proteins were loaded into an SDS-PAGE gel, and then transferred into a PVDF membrane. The membrane was blocked with 5% skim milk for 2h at room temperature and then incubated overnight with the corresponding primary antibody (abs143408, absin) at 4°C. Finally, the membrane was incubated using horseradish peroxidase-conjugated secondary antibodies and exposed by an enhanced chemiluminescence (ECL) kit (Beyotime).

### Immunohistochemistry

The paraffin-embedded specimens were cut into 4 µm thick section for immunohistochemical staining. All sections were dewaxed with xylene and then rehydrated in gradient alcohol. The sections were incubated with 3% H_2_O_2_ at room temperature for 15 minutes and then washed in PBS. Next, 5% BSA was incubated for 1 h at 37°C to block nonspecific binding sites. The sections were incubated with primary antibodies (abs143408, absin) overnight at 4°C. IHC was performed by SP Rabbit & Mouse HRP Kit (DAB) (CWBIO, China).

### Cell culture and plasmid transfection

The human KIRC cell line (769P cells) were maintained at 37°C in a humidified atmosphere containing 5% CO_2_ in RPMI 1640 medium (Gibco, USA) supplemented with 10% fetal bovine serum (Gibco, USA) and 1% penicillin/streptomycin solution (Beyotime, China). According to the manufacturer’s protocol, the plasmids were transfected into 769P cells using lipofectamine_2000 transfection reagent (Invitrogen, USA). After 48 h of transfection, RIPA buffer (Beyotime, China) was used to extract total proteins.

### EdU assay

The EdU assay kit was purchased from Beyotime Company (C0078S, China). According to the manufacturer’s protocol, transfected cells were treated with EdU reagent for 3h. Then, cells were fixed with 4% paraformaldehyde for 15 min at room temperature, followed by incubation with 0.3% Triton X-100 for 15 min at room temperature. Finally, cells were stained with fluorescent dye and Hoechst. The Nikon TI2-D-PD inverted microscope (Japan) was used to capture the images.

### Migration and invasion assay

For the wound healing assay, after 48 hours of transfection, 20 **μ**L of pipette tip was used to scratch the cell plate and the cells were washed by PBS. Then, cells were continued to be cultured with 1% fetal bovine serum (Gibco, USA) at 37°C for 24h. The photographs were taken at 0h and 24h. For the transwell assay, after 48 hours of transfection, 5×10^4^ cells were seeded in a 24-well Transwell chamber (Corning, USA) with a layer of Matrigel matrix glue (Corning, USA) (matrix glue: Serum-free medium=1:6). After 36h, the cells in the upper chamber were fixed with 4% paraformaldehyde for 20 min at room temperature and stain with crystal violet (Sangon Biotech, China) for 20 min. The Nikon TI2-D-PD inverted microscope (Japan) was used to capture the images.

### 5-Azacytidine treatment

5-Azacytidine was purchased from MedChemExpress Company (HY-10586, USA). 769P cell were seeded into six-well plates at appropriate density the day before dosing. After 48h of addition of different concentrations of 5-Azacytidine, the cells were used for subsequent processing. TRIzol (Ambion, USA) was used to extract the total RNA from 769P cells. Then, the HiScript III RT SuperMix for qPCR (+gDNA wiper) (Vazyme, China) was used to perform the reverse transcription reactions. 2X Rapid Taq Master Mix (Vazyme, China) was used to perform gene amplification followed by PCR products added to a 1% agarose gel for electrophoresis. The procedure for protein extraction and western blot were the same as above. The detailed primer sequences are as follows:

GAPDH forward:
5′- TCGACAGTCAGCCGCATCT′ and Reverse: 5′-CTAGCCTCCCGGGTTTCTCT-3′;
OCLN Forward: 5′- ACAAGCGGTTTTATCCAGAGTC′ and Reverse: 5′- GTCATCCACAGGCGAAGTTAAT-3′

### Methylation-specific PCR

After 48h treatment with 5-Azacytidine, genomic DNA was extracted from 769P cells with the FastPure Cell/Tissue DNA Isolation Mini kit (Vazyme, China), according to the manufacturer’s instructions. Next, the DNA(1μg) was treated with EpiArt Magnetic DNA Methylation Bisulfite kit following manufacturer’s instructions (Vazyme, China). The methylated and unmethylated Twist were amplified using 2xEpiArt HS Taq Master Mix (Vazyme, China). The detailed primer sequences were as follows: Methylated: 5′-TTTAAGGTTTTATTCGAAGTAGGC-3′(forward), 5′-GTTACGACCCGAAAAACGAA-3′(reverse); the unmethylated: 5′-TTAAGGTTTTATTTGAAGTAGGTGG-3′(forward), 5′-ACATTACAACCCAAAAAACAAA-3′(reverse). The PCR products were separated in a 1.5% agarose gel.

### Statistical analysis

All data were analyzed using R software (v4.2.2). Univariate and multivariate Cox regression analysis were used to identify independent prognostic factors. All experiments were independently performed at least three times. All statistical analyses were conducted using GraphPad Prism 9.0 (GraphPad, San Diego, CA, USA). The Student’s t-test was used to analyze the differences between two groups, and p<0.05 was considered to be statistically significant.

## Results

### A significant down-regulation of OCLN in KIRC

**Figure 1:**
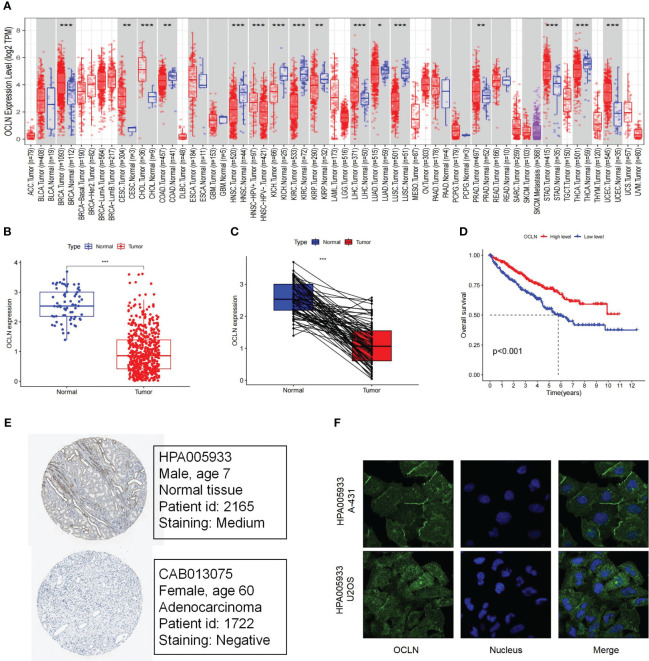
The Expression and prognosis evaluation of OCLN in KIRC. **(A)** The expression of OCLN in pan-cancers. **(B)** The expression of OCLN in 542 KIRC tissues compared with 72 normal renal tissue samples in the TCGA. **(C)** The expression of OCLN in paired KIRC samples from TCGA. **(D)** The survival analyses of OCLN in KIRC. **(E)** The protein expression of OCLN in KIRC from the HPA database. **(F)** Immunofluorescence staining of the subcellular localization of OCLN in HPA database (*: P<0.05; **: P<0.01; ***: P<0.001).

By TIMER database mining, the expression level of OCLN was obtained from a pan-cancer perspective. As shown in **Figure 1A**, the expression of OCLN was down-regulated in eight cancer types including KIRC. In **Figures 1B, C**, OCLN mRNA expression was significantly down-regulated in KIRC compared to normal tissues from the TCGA dataset. Kaplan-Meier survival curves showed that low OCLN expression was correlated with poor prognosis for KIRC patients (**Figure 1D**). Moreover, the protein-level expression of OCLN shown in **Figure 1E** was also down-regulated in KIRC according to the records in HPA database (28), and OCLN subcellular localization was obtained by immunofluorescence localization of the plasma membrane and cell junctions in A-431 and U2OS cells (**Figure 1F**). Overall, the analysis by public data sources summarized that OCLN was significantly downregulated in KIRC both at the mRNA and protein level.

### Low expression of OCLN correlated with poor clinicopathological features

**Figure 2:**
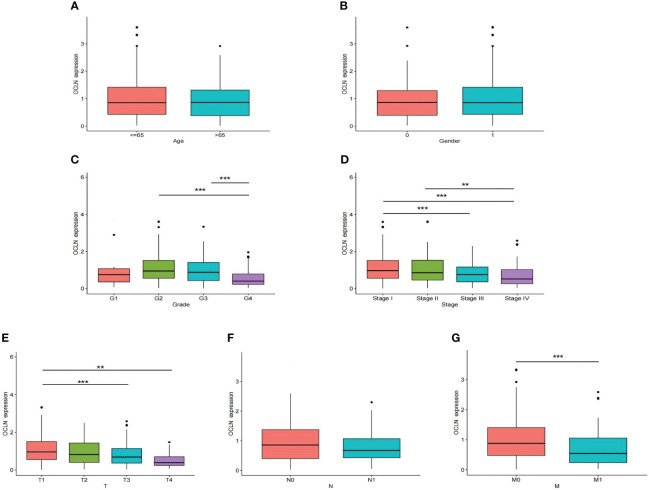
The correlation of OCLN expression with clinicopathologic features in the TCGA-KIRC cohort. **(A)** age. **(B)** gender. **(C)** tumor grade. **(D)** clinical stage. **(E)** T stage. **(F)** N stage. **(G)** M stage (**: P<0.01; ***: P<0.001).

The correlation between OCLN expression and corresponding clinicopathological features was analyzed to explore the clinical role of OCLN in KIRC. As shown in **Figures 2A–G**, the expression of OCLN was significantly related to tumor grade, clinical stage, T stage, and M stage in KIRC patients. The lower expression of OCLN was correlated with the worse pathological grade, later clinical stage, higher T stage and M stage in KIRC. The results indicated that low expression of OCLN may promote proliferation and metastasis of KIRC, resulting in a poor prognosis for KIRC patients.

### Promoter methylation levels of OCLN in different KIRC groups

**Figure 3:**
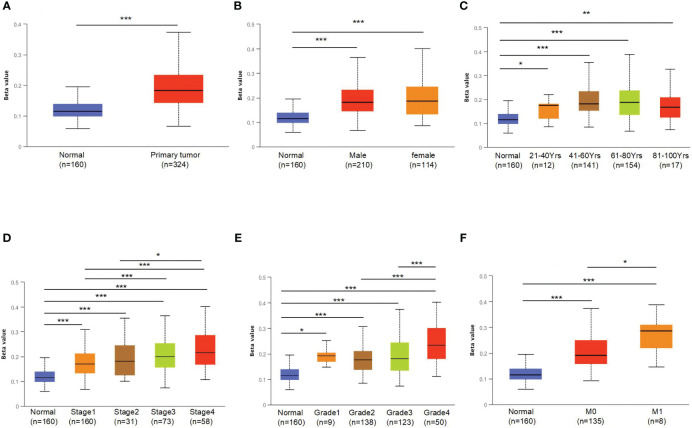
The promoter methylation levels of OCLN in different patient groups. **(A)** Promoter methylation levels of OCLN were high in KIRC. **(B)** gender. **(C)** age. **(D)** clinical stage. **(E)** tumor grade. **(F)** N stage (*: P<0.05; **: P<0.01; ***: P<0.001).

To further explore the underlying mechanism of OCLN low-expression in KIRC, DNA methylation analysis was performed. As known, DNA methylation is an important event in the epigenetic modification of the genome and is closely related to disease process. The DNA methylation levels of OCLN for KIRC were tested using the UALCAN database. As shown in **Figures 3A, B**, the methylation level of OCLN in KIRC was higher than that in normal tissues, but there was no difference between the gender groups. Moreover, the methylation level of OCLN was positively correlated with age, clinical stage, and tumor grade level (**Figures 3C–E**). As N stage increased, the methylation level of OCLN tended to be highly upregulated (**Figure 3F**). The results suggested that DNA hypermethylation may be one of the potential reasons for low-expression of OCLN in KIRC.

### OCLN as an independent prognostic factor for KIRC

**Figure 4:**
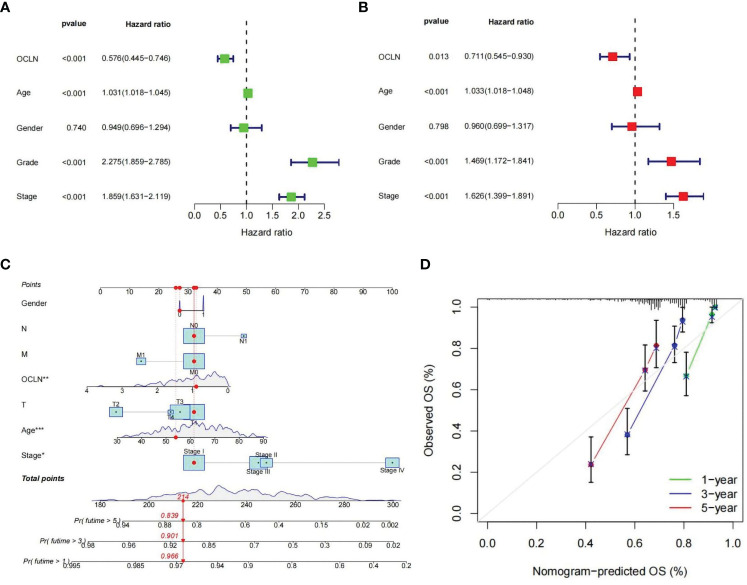
Construction of the OCLN-based nomogram. **(A)** Univariate Cox regression analysis of clinical factors and OCLN expression for overall survival. **(B)** Multivariate Cox regression analysis of clinical factors and OCLN expression for overall survival. **(C)** Nomogram construction. **(D)** Calibration curves of nomogram for 1, 3 and 5 years.

To further evaluate the prognostic value of OCLN in KIRC, univariate and multivariate Cox regression were performed. The univariate Cox analysis indicated that OCLN expression (p<0.001), age (p<0.001), grade (p<0.001), and stage (p<0.001) were significant factors for overall survival of KIRC patients (**Figure 4A**). Multivariate Cox regression analysis further identified that the expression of OCLN (p=0.013) was an independent prognostic factor (**Figure 4B**). Based on these findings, a novel nomogram with OCLN expression and clinical parameters was constructed to predict 1-,3- and 5-year OS for KIRC patients (**Figure 4C**), and a higher score on the nomogram indicated a worse prognosis for a KIRC patient. As shown in **Figure 4D**, the nomogram calibration curve had good reliability in the observation of 1-, 3-, and 5- year survival rate.

### OCLN potentially regulated immune process in KIRC development

**Figure 5:**
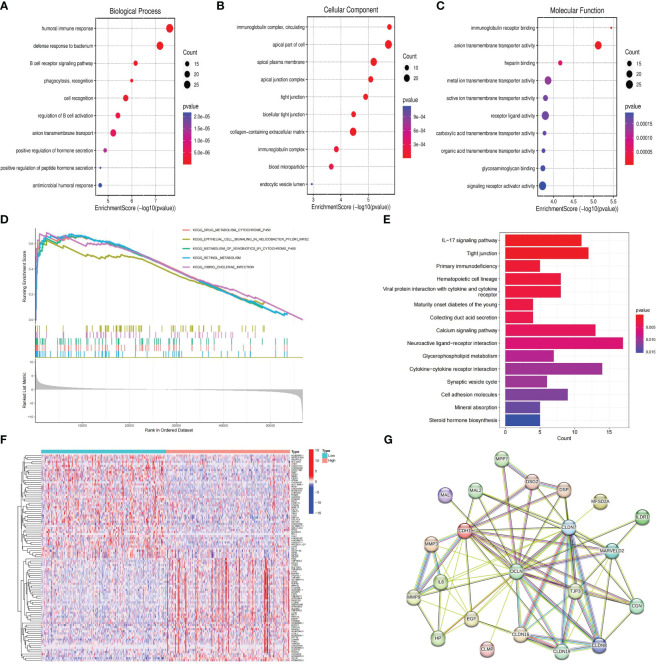
Functional enrichment and protein-protein interaction network analysis of OCLN in KIRC. **(A)** GO-BP enrichment analysis. **(B)** GO-CC enrichment analysis. **(C)** GO-MF enrichment analysis. **(D)** GSEA analysis of OCLN. **(E)** KEGG analysis of pathways related to DEGs. **(F)** The heatmap of top 50 up-regulated and down-regulated DEGs. **(G)** OCLN-related protein-protein interactions.

Patients with KIRC in TCGA were divided into high and low risk groups according to the median value of OCLN expression to explore the underlying molecular function of OCLN. A total of 834 DEGs were identified based on differential expression analysis between high and low risk groups, among which 335 genes were upregulated and 499 genes were downregulated (|log_2_FC |> 1 and FDR < 0.01, **Table S2**). As shown in **Figures 5A–C**, GO enrichment analysis at biological process (BP), cellular component (CC), and molecular function (MF) levels showed that DEGs were involved in immune-related functions. For example, in GO-BP domain, the genes were enriched in humoral immune response, B cell receptor signaling pathway and regulation of B cell activation. In GO-CC domain, the genes were associated with immunoglobulin complex circulating and immunoglobulin complex. In GO-MF domain, immunoglobulin receptor binding was observed to be functionally enriched. As shown in **Figures 5D, E**, GSEA enrichment analysis indicated that the drug metabolism cytochrome P450, epithelial cell signaling in helicobacter pylori infection and metabolism of xenobiotics by cytochrome P450 were upregulated in the low OCLN expression group, and the DEGs were significantly enriched in KEGG pathways such as IL-17 signaling pathway, Tight junction and primary immunodeficiency.

To further decipher the potential interplay among OCLN and DEGs, an OCLN-related protein-protein interaction network was constructed and analyzed. As shown in **Figures 5F, G**, the top 50 up- and down-regulated genes between OCLN high- and low-expression group were clustered, and a total of 21 genes with direct links with OCLN were visualized by STRING online. Through correlation analysis in the GEPIA (29) database, a total of 14 genes, i.e., CDH1, CGN, CLDN7, CLDN8, CLDN16, CLDN19, DSP, EGF, ILDR1, MAL, MAL2, MARVELD2, MPP7 and TJP3, were determined to be highly correlated with OCLN (R>0.5; P<0.05) (**Figure S1**).

### OCLN expression was associated with immune signatures in KIRC

**Figure 6:**
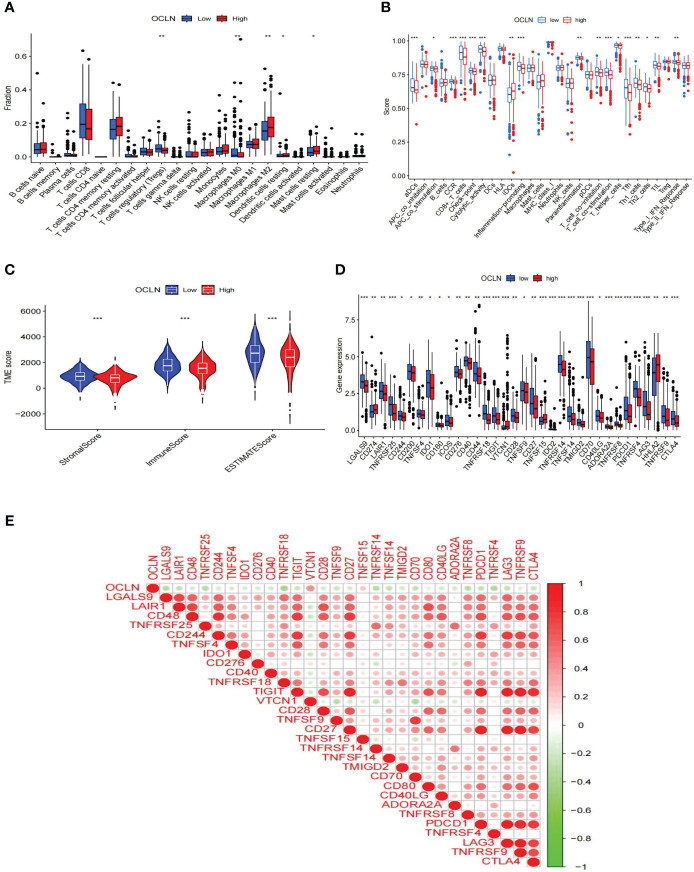
Correlations of OCLN expression with immune infiltration level in KIRC. **(A)** Histogram of immune cell infiltration between high- and low-OCLN groups. **(B)** Histogram of immune function between high- and low-OCLN groups. **(C)** The association between the two groups in Immune scores, Stromal scores, and ESTIMATE scores. **(D)** The expression of immune checkpoint genes between high- and low-OCLN groups. **(E)** Relationship between OCLN expression and immune checkpoint genes in KIRC. *: P<0.05; **: P<0.01; ***: P<0.001.

The role of OCLN in the immune microenvironment of KIRC was explored using the ESTIMATE method to analyze the correlation between OCLN expression and immune cell infiltration. As shown in **Figure 6A**, the results showed that the low-OCLN-expression group had higher levels of infiltration of Tregs and Macrophages M0 cells than the low-OCLN-expression group. The effects of OCLN expression on the OS of KIRC patients with high or low immune cell infiltration were also explored using Kaplan-Meier Plotter database. The high expression of OCLN of KIRC in enriched basophils, enriched macrophages, decreased mesenchymal stem cells and decreased Type 1 T-helper cells had better prognosis (**Figure S2**). It suggested that high expression of OCLN in KIRC may affect prognoses partly due to immune cells infiltration. As observed in **Figure 6B**, the low OCLN-expression group revealed higher scores in multiple immune functions, such as aDCs, APC_co_stimulation, CCR, CD8^+^T cells, Inflammation-promoting, Parainflammation, T_cell_co_inhibition, TIL, Tfh,checkpoint, Cytolytic activity and Type_I_IFN_Reponse. The ESTIMATE algorithm was also applied to calculate stromal score, immune score, and ESTIMATE score. As shown in **Figure 6C**, the lower OCLN level was associated with higher stromal score, immune score and ESTIMATE score. In addition, the interactions between OCLN and immune checkpoint genes were evaluated. As shown in **Figure 6D**, the majority of immune checkpoint genes increased significantly in the OCLN low group, such as CTLA4 and PDCD1, and OCLN was negatively associated with overwhelming majority immune checkpoint molecules (**Figure 6E**). In summary, OCLN played potential roles in immune escape of KIRC and could serve as a candidate target for KIRC immunotherapy.

### Experimental verification

**Figure 7:**
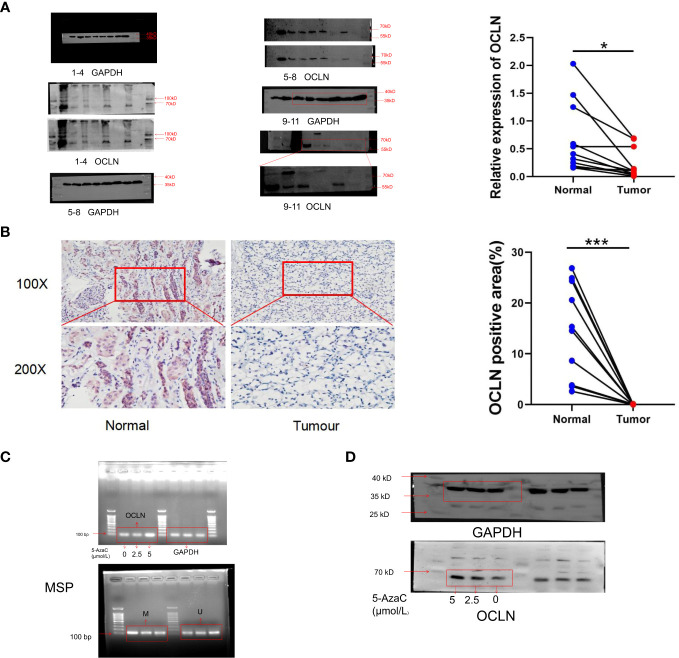
Promoter hypermethylation resulted in downregulation of OCLN in KIRC. **(A)** Immunoblotting analysis of OCLN expression between KIRC and adjacent normal renal tissues. The Image J software was used to analyze the grayscale value of protein bands. Original image of blots/gels are presented in **Supplementary Figure S3**. **(B)** Immunohistochemistry staining of OCLN in KIRC and adjacent normal renal tissue. The Image J software was used for quantitative analysis. **(C)** OCLN mRNA upregulated and promoter demethylation in 769P cells after 5-Azac treatment. **(D)** OCLN protein expression in 769P cells after 5-Azac demethylation treatment (*: P<0.05; ***: P<0.001). Original image of blots/gels are presented in **Supplementary Figure S4**.

Western blot and immunohistochemistry (IHC) experiments were performed respectively to validate the expression of OCLN in clinical samples. As shown in **Figures 7A, B**, the protein expression of OCLN was decreased in KIRC tissue samples, and the immunohistochemistry staining further verified that the expression of OCLN was lower in tumor tissues. 5-azacytidine(5-Azac) is a DNA methylation inhibitor. To investigate the effect of promoter methylation on OCLN gene expression, 769P cells was treated with 5-Azac at concentrations of 0 μM, 2.5 μM, and 5 μM, respectively. As shown in **Figures 7C, D**, treated with 48 hours of 5-azacytidine induced demethylation of the OCLN promoter and increased OCLN mRNA and protein expression in 769P cells.

**Figure 8:**
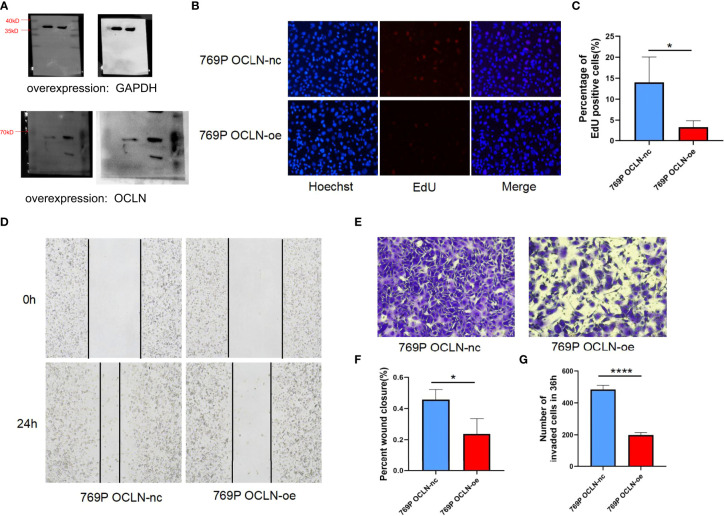
Functional verification of OCLN in 769P cells. **(A)** Verification of overexpression efficiency of OCLN in 769P cells. Original image of blots/gels are presented in **Supplementary Figure S3**. **(B, C)** Representative images of the EdU assay in 769P cells with OCLN overexpression. **(D)** OCLN overexpression inhibits migration in 769P cells. **(E)** OCLN overexpression inhibits invasion in 769P cells. **(F)** The Image J software was used to calculate the area of wound healing. **(G)** The Image J software was used to calculate the number of invaded cells (*: P<0.05; ****: P<0.0001).

Moreover, 769P cell line was selected for follow-up functional experiments to confirm the results of bioinformatics analysis. As shown in **Figures 8A–C**. OCLN was successfully overexpressed in 769P cells, and the overexpression of OCLN significantly inhibited cell proliferation as indicated by EdU assays. Meanwhile, as shown in **Figures 8D–G**, OCLN overexpression significantly reduced the migration and invasion abilities of 769P cells.

## Discussion

Kidney cancer is the third most common urinary malignancies, and the clear cell carcinoma is often observed as a typical pathological subtype. Surgical resection is the ideal method for treatment of KIRC patients, unfortunately a great number of patients are suffered from tumor recurrence after surgery (30, 31). Currently, immunotherapy is an emerging direction for KIRC treatment (32, 33). Therefore, finding new biomarkers associated with the immunomodulation is of significance for KIRC precision and personalized medicine.

OCLN is known to be presented in all the tight junctions. The expression of OCLN in KIRC was significantly lower than that in normal renal tissues in this study. Generally, the downregulation of tumor suppressor genes expression may be due to transcriptional repression, promoter methylation, or increased protein degradation. Mo et al. demonstrated that calprotectin subunit S100A9 can semimethylate the promoter of OCLN in melanoma cells, resulting in reduced expression of OCLN (34). In this study, the methylation level of OCLN promoters in KIRC was significantly higher than that in normal renal tissues. Moreover, the methylation level of OCLN was increased with age, tumor grade, clinical stage and N stage. The results of *in vitro* experiments showed that the expression of OCLN increased significantly when the methylation level in KIRC cells was inhibited. The low expression of OCLN was associated with tumorigenesis and progression of KIRC. In the TCGA-KIRC clinical cohort, the expression level of OCLN was negatively related to tumor grade, clinical stage, T stage and M stage. In addition, KIRC patients with lower OCLN expression had a poorer prognosis. Univariate and multivariate analysis showed that OCLN was an independent prognostic factor. *In vitro* experiments showed that OCLN overexpression inhibited the proliferation, migration and invasion of KIRC cells. Therefore, these findings indicated that OCLN may act as a tumor suppressor for KIRC.

To investigate the underlying molecular mechanism of OCLN in KIRC carcinogenesis, an OCLN-related protein-protein interaction network was constructed and the correction analysis was performed. Based on network modeling, a total of 14 genes were highly relevant to OCLN, and some of them have been reported to be strongly associated with the development of KIRC. For example. Li et al. demonstrated that CLDN7 expression was reduced in KIRC, and overexpression of CLDN7 inhibited the proliferation, migration and invasion ability of KIRC cells *in vitro* and *in vivo (* 14). MPP7 is a protein coding gene and the protein encoded by this gene is a member of the membrane-associated guanylate kinase (MAGUK) protein p55 Stardust family. Long et al. demonstrated that miR-421 downregulation inhibited the malignant phenotype of KIRC by increasing MPP7 levels (35). It could be supposed that OCLN may affect the formation and progression of KIRC directly or indirectly by interacting with known KIRC-associated genes for KIRC phenotype regulation.

The tumor microenvironment is composed of tumor cells, a variety of stromal cells, cytokines, chemokines, etc. (36–38). It is acknowledged that tumor-infiltrating immune cells, as one of the components of the tumor microenvironment, were closely linked to the progression and metastasis of tumor (39, 40). The function of immune cells in the tumor microenvironment is either enhanced or weakened, creating an immunosuppressive environment that contribute to immune escape and drug resistance. In this study, the scores of immune cells and immune stromal in the OCLN low-expression group were significantly higher than that in the OCLN high-expression group, indicating that there was more immune cell infiltration in the OCLN low-expression group. Meanwhile, the difference in the distribution of immune cells between the high OCLN expression group and low OCLN expression group also showed that Tregs cells and M0 macrophages were more enriched in the low OCLN expression group. Tregs cells are a subpopulation of T cells with significant immunosuppressive effects, which could suppress the immune response of other cells and in turn lead to tumor proliferation and metastasis (41, 42). Significant invasion of Treg cells in the low OCLN expression group could led to KIRC progression by suppressing the immune response, which was consistent with the poor prognosis of the low OCLN expression group. GO and KEGG enrichment analysis indicated that OCLN may be involved in a variety of immune activities such as humoral immune response, B cell receptor signaling pathway, immunoglobulin complex circulating and primary immunodeficiency. Hence, the differences in immune-related functions between OCLN high- and low-expression groups were compared, and significant differences between the OCLN high- and low-expression groups at immune checkpoints were evaluated. Tumor cells expressed PD-L1 ligands that match the T-cell PD-1 protein, preventing them from finding the tumor and sending signals to the immune system to attack the tumor, directly leading to T-cell failure (43, 44). KIRC patients with distant metastasis and recurrence after surgery often had poor prognosis. Currently, immune checkpoint inhibitors (ICIs) have delighted new direction for advanced KIRC patients. However, only a small percentage of patients respond to immunotherapy, highlighting the urgency for identification of sensitive prognostic biomarkers to improve treatment response. In this study, OCLN expression was found to be significantly negatively correlated with PDCD1 and CTLA4 expression, which implicated the role of OCLN in regulating tumor immunology in KIRC. The expression of OCLN could be used to predict the clinical response of ICI, thereby contributing to the personalized therapy for KIRC patients.

In summary, this study deciphered the role of OCLN in KIRC clinical characteristics, prognosis, immunotherapy. OCLN could be considered as a potential biomarker for KIRC personalized medicine. It should be concerned that several limitations still need to be considered. First, the conclusions were not confirmed by *in vivo* experiments. Second, the mechanism between OCLN expression and the infiltration of immune cells was not well explained. More clinical and pathogenic experiments will be conducted in the future work to investigate the carcinogenesis of OCLN during KIRC evolution.

## Conclusions

This study showed that OCLN was downregulated in KIRC, and the low OCLN expression was associated with poor prognosis in KIRC. Moreover, the expression of OCLN was associated with immune cell infiltration and could act as a novel potential biomarker and therapeutic target for KIRC prognosis and immunotherapy. Further experiments need to be conducted to explore the molecular mechanisms of OCLN in KIRC.

## Data availability statement

The original contributions presented in the study are included in the article/**Supplementary Material**. Further inquiries can be directed to the corresponding authors.

## Ethics statement

The studies involving humans were approved by the ethics committee of The First Affiliated Hospital of Soochow University. The studies were conducted in accordance with the local legislation and institutional requirements. The participants provided their written informed consent to participate in this study.

## Author contributions

ZJ, YK, and CW contributed equally to this study. YL and YH designed and supervised the study jointly. ZJ, ZF, ZT, YS and YL performed and validated the bioinformatics analysis. ZJ, YK, CW and YH designed the experimental scheme and performed the experiments. ZJ, YK, CW, YL and YH drafted and revised this manuscript. YL and YH provided support for publishing funds. All authors contributed to the article and approved the submitted version.

## Supplementary material

Supplementary Figure S3 Original image of blots/gels for **Figures 7A**, **8A**.

Supplementary Figure S4 Original image of blots/gels for **Figures 7C, 7D**.

## References

[1] Ferlay J Colombet M Soerjomataram I Dyba T Randi G Bettio M. Cancer incidence and mortality patterns in Europe: Estimates for 40 countries and 25 major cancers in 2018. Eur J Cancer (2018) 103:356–87. doi: 10.1016/j.ejca.2018.07.005 30100160

[2] Capitanio U Bensalah K Bex A Boorjian SA Bray F Coleman J. Epidemiology of renal cell carcinoma. Eur Urol (2019) 75(1):74–84. doi: 10.1016/j.eururo.2018.08.036 30243799 PMC8397918

[3] Nabi S Kessler ER Bernard B Flaig TW Lam ET. Renal cell carcinoma: a review of biology and pathophysiology. F1000Res (2018) 7:307. doi: 10.12688/f1000research.13179.1 29568504 PMC5850086

[4] Flanigan RC Mickisch G Sylvester R Tangen C Van Poppel H Crawford ED. Cytoreductive nephrectomy in patients with metastatic renal cancer: a combined analysis. J Urol (2004) 171(3):1071–6. doi: 10.1097/01.ju.0000110610.61545.ae 14767273

[5] Bex A Albiges L Ljungberg B Bensalah K Dabestani S Giles RH. Updated European association of urology guidelines for cytoreductive nephrectomy in patients with synchronous metastatic clear-cell renal cell carcinoma. Eur Urol (2018) 74(6):805–9. doi: 10.1016/j.eururo.2018.08.008 30177291

[6] Norberg SM Hinrichs CS. Engineered T cell therapy for viral and non-viral epithelial cancers. Cancer Cell (2023) 41(1):58–69. doi: 10.1016/j.ccell.2022.10.016 36400016 PMC9839504

[7] Soerens AG Künzli M Quarnstrom CF Scott MC Swanson L Locquiao JJ. Functional T cells are capable of supernumerary cell division and longevity. Nature (2023) 614(7949):762–6. doi: 10.1038/s41586-022-05626-9 PMC11617068 36653453

[8] Bell HN Huber AK Singhal R Korimerla N Rebernick RJ Kumar R. Microenvironmental ammonia enhances T cell exhaustion in colorectal cancer. Cell Metab (2023) 35(1):134–149.e136. doi: 10.1016/j.cmet.2022.11.013 36528023 PMC9841369

[9] Flippot R Escudier B Albiges L. Immune checkpoint inhibitors: toward new paradigms in renal cell carcinoma. Drugs (2018) 78(14):1443–57. doi: 10.1007/s40265-018-0970-y 30187355

[10] Motzer RJ Tannir NM McDermott DF Arén Frontera O Melichar B Choueiri TK. Nivolumab plus Ipilimumab versus Sunitinib in Advanced Renal-Cell Carcinoma. N Engl J Med (2018) 378(14):1277–90. doi: 10.1056/NEJMoa1712126 PMC5972549 29562145

[11] Hsieh JJ Purdue MP Signoretti S Swanton C Albiges L Schmidinger M. Renal cell carcinoma. Nat Rev Dis Primers (2017) 3:17009. doi: 10.1038/nrdp.2017.9 28276433 PMC5936048

[12] Monteiro FSM Soares A Rizzo A Santoni M Mollica V Grande E. The role of immune checkpoint inhibitors (ICI) as adjuvant treatment in renal cell carcinoma (RCC): A systematic review and meta-analysis. Clin Genitourin Cancer (2023) 21(3):324–33. doi: 10.1016/j.clgc.2023.01.005 36823017

[13] Owari T Sasaki T Fujii K Fujiwara-Tani R Kishi S Mori S. Role of nuclear claudin-4 in renal cell carcinoma. Int J Mol Sci (2020) 21(21):8340. doi: 10.3390/ijms21218340 33172177 PMC7664319

[14] Li Y Gong Y Ning X Peng D Liu L He S. Downregulation of CLDN7 due to promoter hypermethylation is associated with human clear cell renal cell carcinoma progression and poor prognosis. J Exp Clin Cancer Res (2018) 37(1):276. doi: 10.1186/s13046-018-0924-y 30428910 PMC6234584

[15] Yang F Liu XQ He JZ Xian SP Yang PF Mai ZY. Occludin facilitates tumour angiogenesis in bladder cancer by regulating IL8/STAT3 through STAT4. J Cell Mol Med (2022) 26(8):2363–76. doi: 10.1111/jcmm.17257 PMC8995457 35224833

[16] Pudova EA Kobelyatskaya AA Katunina IV Snezhkina AV Fedorova MS Pavlov VS. Lymphatic dissemination in prostate cancer: features of the transcriptomic profile and prognostic models. Int J Mol Sci (2023) 24(3):2418. doi: 10.3390/ijms24032418 36768739 PMC9916851

[17] Murakami T Felinski EA Antonetti DA. Occludin phosphorylation and ubiquitination regulate tight junction trafficking and vascular endothelial growth factor-induced permeability. J Biol Chem (2009) 284(31):21036–46. doi: 10.1074/jbc.M109.016766 PMC2742868 19478092

[18] Li T Fan J Wang B Traugh N Chen Q Liu JS. TIMER: A web server for comprehensive analysis of tumor-infiltrating immune cells. Cancer Res (2017) 77(21):e108–10. doi: 10.1158/0008-5472.Can-17-0307 PMC6042652 29092952

[19] Uhlén M Fagerberg L Hallström BM Lindskog C Oksvold P Mardinoglu A. Proteomics. Tissue-based map of the human proteome. Science (2015) 347(6220):1260419. doi: 10.1126/science.1260419 25613900

[20] Chandrashekar DS Bashel B Balasubramanya SAH Creighton CJ Ponce-Rodriguez I Chakravarthi B. UALCAN: A portal for facilitating tumor subgroup gene expression and survival analyses. Neoplasia (2017) 19(8):649–58. doi: 10.1016/j.neo.2017.05.002 PMC5516091 28732212

[21] Gene Ontology Consortium Harris MA Clark JI Ireland A Lomax J Ashburner M.The gene ontology (GO) project in 2006. Nucleic Acids Res (2006) 34(Database issue):D322–326. doi: 10.1093/nar/gkj021 PMC1347384 16381878

[22] Kanehisa M Goto S. KEGG: kyoto encyclopedia of genes and genomes. Nucleic Acids Res (2000) 28(1):27–30. doi: 10.1093/nar/28.1.27 10592173 PMC102409

[23] Yu G Wang LG Han Y He QY. clusterProfiler: an R package for comparing biological themes among gene clusters. Omics (2012) 16(5):284–7. doi: 10.1089/omi.2011.0118 PMC3339379 22455463

[24] Powers RK Goodspeed A Pielke-Lombardo H Tan AC Costello JC. InContext: identifying novel and common patterns in expression experiments. Bioinformatics (2018) 34(13):i555–64. doi: 10.1093/bioinformatics/bty271 PMC6022535 29950010

[25] Szklarczyk D Gable AL Lyon D Junge A Wyder S Huerta-Cepas J. STRING v11: protein-protein association networks with increased coverage, supporting functional discovery in genome-wide experimental datasets. Nucleic Acids Res (2019) 47(D1):D607–d613. doi: 10.1093/nar/gky1131 30476243 PMC6323986

[26] Eng KH Schiller E Morrell K. On representing the prognostic value of continuous gene expression biomarkers with the restricted mean survival curve. Oncotarget (2015) 6(34):36308–18. doi: 10.18632/oncotarget.6121 PMC4742179 26486086

[27] Gentles AJ Newman AM Liu CL Bratman SV Feng W Kim D. The prognostic landscape of genes and infiltrating immune cells across human cancers. Nat Med (2015) 21(8):938–45. doi: 10.1038/nm.3909 PMC4852857 26193342

[28] Asplund A Edqvist PH Schwenk JM Pontén F. Antibodies for profiling the human proteome-The Human Protein Atlas as a resource for cancer research. Proteomics (2012) 12(13):2067–77. doi: 10.1002/pmic.201100504 22623277

[29] Tang Z Li C Kang B Gao G Li C Zhang Z. GEPIA: a web server for cancer and normal gene expression profiling and interactive analyses. Nucleic Acids Res (2017) 45(W1):W98–w102. doi: 10.1093/nar/gkx247 28407145 PMC5570223

[30] Capitanio U Montorsi F. Identifying patients for adjuvant therapy after nephrectomy. Lancet (2022) 400(10358):1080–1. doi: 10.1016/s0140-6736(22)01747-0 36099928

[31] Arai T Sazuka T Sato H Kato M Kamada S Katsura S. A clinical investigation of recurrence and lost follow-up after renal cell carcinoma surgery: a single-center, long-term, large cohort, retrospective study. Int J Clin Oncol (2022) 27(9):1467–76. doi: 10.1007/s10147-022-02204-x 35767079

[32] Longhitano E Muscolino P Lo Re C Ferrara SA Cernaro V Gembillo G. Immune checkpoint inhibitors and the kidney: A focus on diagnosis and management for personalised medicine. Cancers (Basel) (2023) 15(6):1891. doi: 10.3390/cancers15061891 36980777 PMC10046877

[33] Hont AB Dumont B Sutton KS Anderson J Kentsis A Drost J. The tumor microenvironment and immune targeting therapy in pediatric renal tumors. Pediatr Blood Cancer (2023) 70(Suppl 2):e30110. doi: 10.1002/pbc.30110 36451260

[34] Najafi M Alizadeh SA Azad M Naserpour Farivar T Rajaei F Hotam Sorouri K. Effect of calprotectin subunit S100A9 on the expression and methylation of OCLN in human melanoma cell line A-375. Turk J Biol (2017) 41(6):849–56. doi: 10.3906/biy-1704-14 PMC6353305 30814850

[35] Pei L Lv X Jia G Liang X Song X Zhang A. CircSCNN1A is a tumor suppressor in renal cell carcinoma via inducing the upregulation of MPP7 by the sponge effect on miR-421. Transpl Immunol (2022) 75:101736. doi: 10.1016/j.trim.2022.101736 36343886

[36] Vitale I Manic G Coussens LM Kroemer G Galluzzi L. Macrophages and metabolism in the tumor microenvironment. Cell Metab (2019) 30(1):36–50. doi: 10.1016/j.cmet.2019.06.001 31269428

[37] Arneth B. Tumor microenvironment. Medicina (Kaunas) (2019) 56(1):15. doi: 10.3390/medicina56010015 31906017 PMC7023392

[38] Wu T Dai Y. Tumor microenvironment and therapeutic response. Cancer Lett (2017) 387:61–8. doi: 10.1016/j.canlet.2016.01.043 26845449

[39] Aponte-López A Muñoz-Cruz S. Mast cells in the tumor microenvironment. Adv Exp Med Biol (2020) 1273:159–73. doi: 10.1007/978-3-030-49270-0_9 33119881

[40] Pan Y Yu Y Wang X Zhang T. Tumor-associated macrophages in tumor immunity. Front Immunol (2020) 11:583084. doi: 10.3389/fimmu.2020.583084 33365025 PMC7751482

[41] Iglesias-Escudero M Arias-González N Martínez-Cáceres E. Regulatory cells and the effect of cancer immunotherapy. Mol Cancer (2023) 22(1):26. doi: 10.1186/s12943-023-01714-0 36739406 PMC9898962

[42] Tanaka A Sakaguchi S. Targeting Treg cells in cancer immunotherapy. Eur J Immunol (2019) 49(8):1140–6. doi: 10.1002/eji.201847659 31257581

[43] Yi M Zheng X Niu M Zhu S Ge H Wu K. Combination strategies with PD-1/PD-L1 blockade: current advances and future directions. Mol Cancer (2022) 21(1):28. doi: 10.1186/s12943-021-01489-2 35062949 PMC8780712

[44] Jiang Y Chen M Nie H Yuan Y. PD-1 and PD-L1 in cancer immunotherapy: clinical implications and future considerations. Hum Vaccin Immunother (2019) 15(5):1111–22. doi: 10.1080/21645515.2019.1571892 PMC6605868 30888929

