# Supplementary material for: OCLN as a novel biomarker for prognosis and immune infiltrates in kidney renal clear cell carcinoma: an integrative computational and experimental characterization
Source: Front Immunol. 2023 Sep 22;14:1224904. doi: 10.3389/fimmu.2023.1224904 (PMC10556524; doi:10.3389/fimmu.2023.1224904)
Supplement: Supplementary Figure S3 — Original image of blots/gels for Figures 7A , 8A . [file DataSheet_1.zip › Figure S1.PDF]

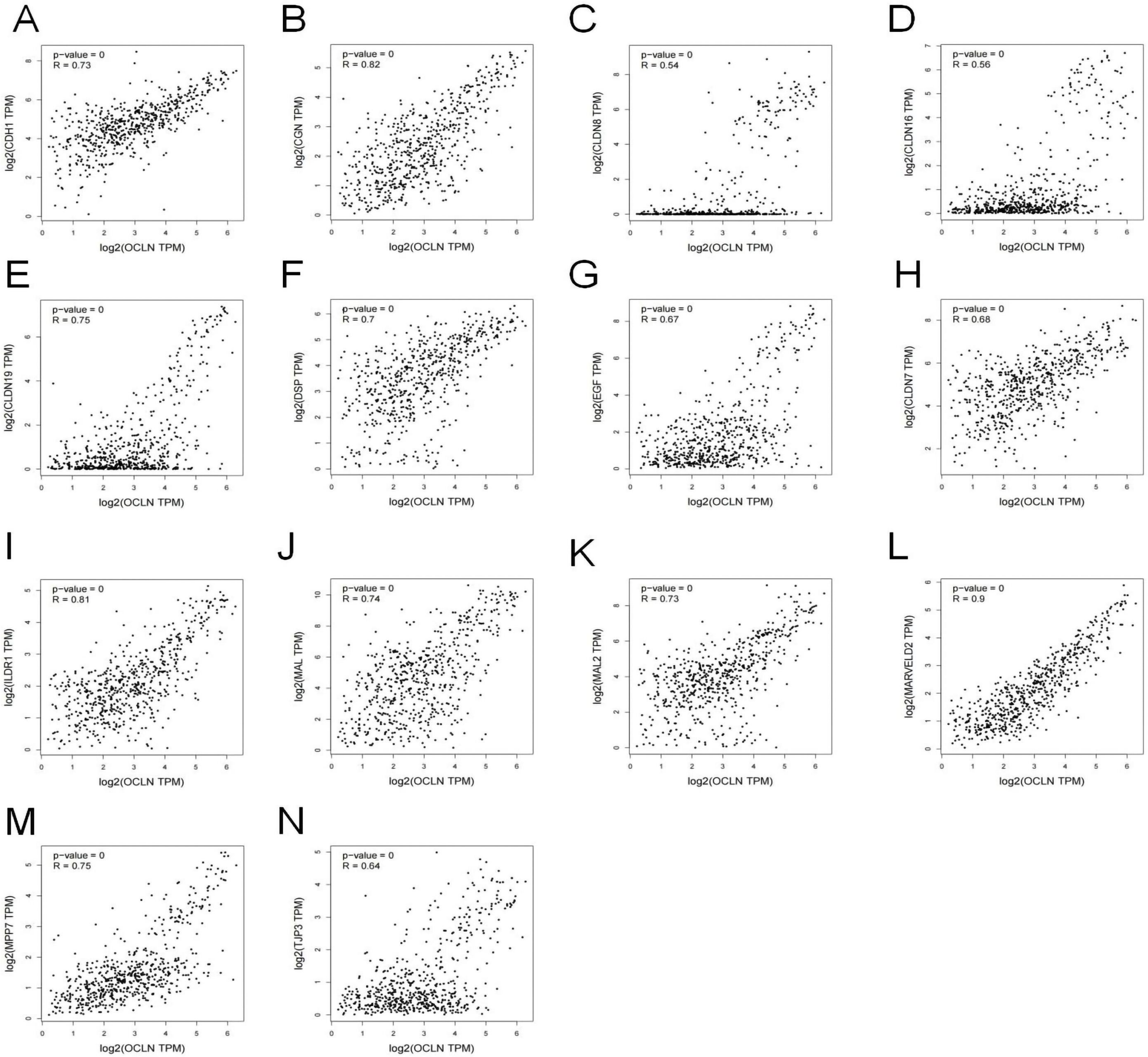

Figure S1. OCLN-related correlation analysis: **(A)** CDH1; **(B)** CGN; **(C)** CLDN8; **(D)** CLDN16; **(E)** CLDN19; **(F)** DSP; **(G)** EGF; **(H)** CLDN7; **(I)** ILDR1; **(J)** MAL; **(K)** MAL2; **(L)** MARVELD2; **(M)** MPP7; **(N)** TJP3.
