# Supplementary material for: OCLN as a novel biomarker for prognosis and immune infiltrates in kidney renal clear cell carcinoma: an integrative computational and experimental characterization
Source: Front Immunol. 2023 Sep 22;14:1224904. doi: 10.3389/fimmu.2023.1224904 (PMC10556524; doi:10.3389/fimmu.2023.1224904)
Supplement: Supplementary Figure S3 — Original image of blots/gels for Figures 7A , 8A . [file DataSheet_1.zip › Figure S2.PDF]

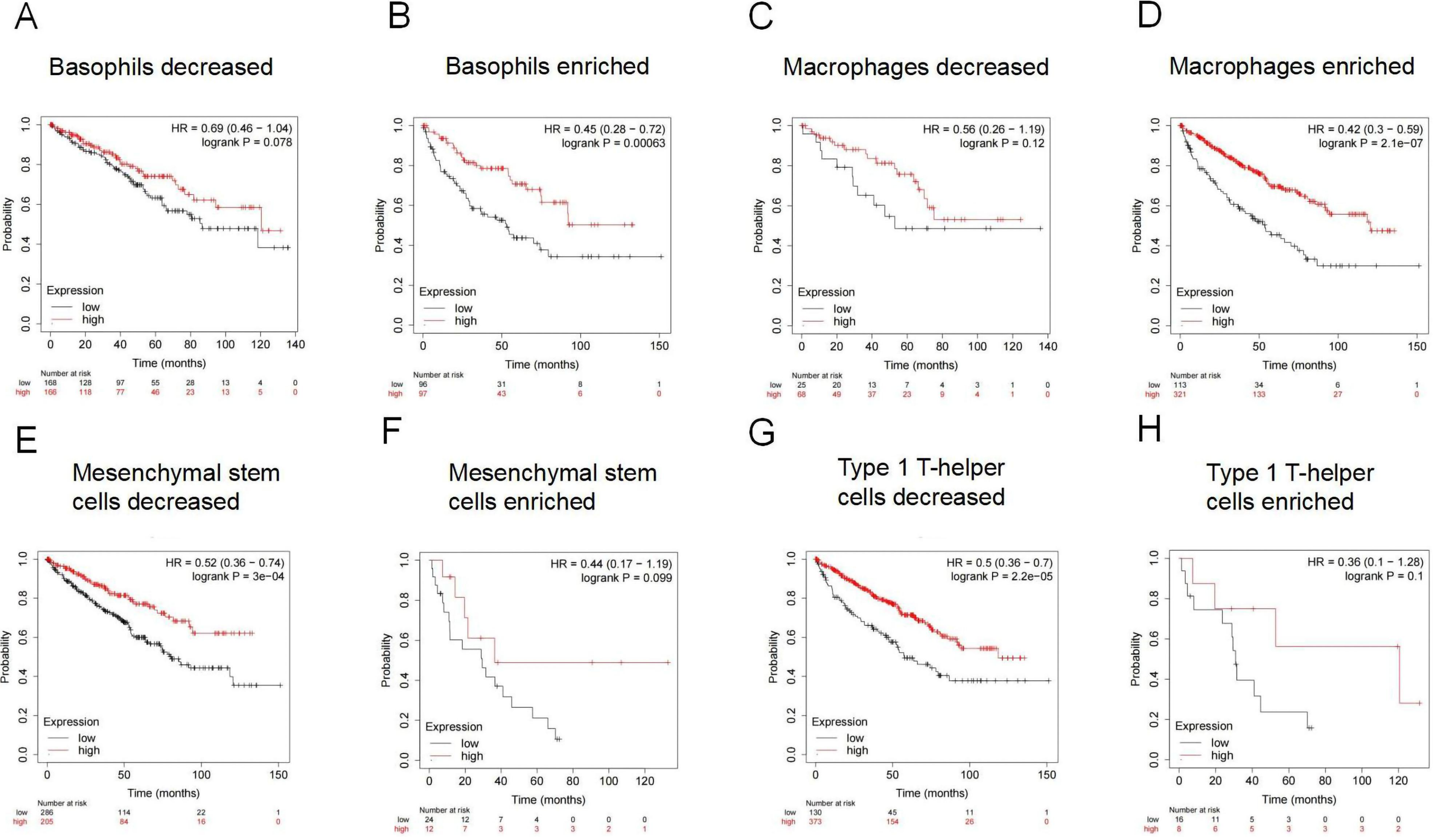

Figure S2. The influence of OCLN expression on the OS of KIRC patients with immune cells subgroups. **(A, B)** Basophils. **(C, D)** Macrophages. **(E, F)** Mesenchymal stem cells. **(F, G)** Type 1 T-helper cells.
