# Supplementary figures and images for: OCLN as a novel biomarker for prognosis and immune infiltrates in kidney renal clear cell carcinoma: an integrative computational and experimental characterization
Source: Front Immunol. 2023 Sep 22;14:1224904. doi: 10.3389/fimmu.2023.1224904 (PMC10556524; doi:10.3389/fimmu.2023.1224904)

Figure 7A

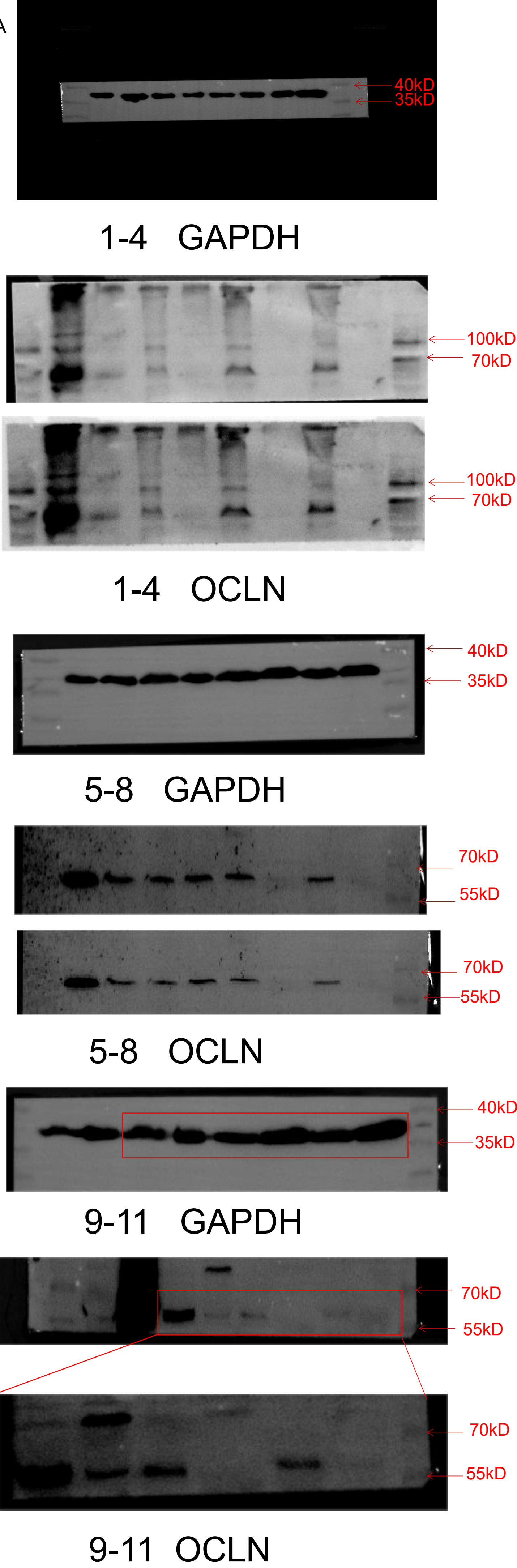

Figure 8A

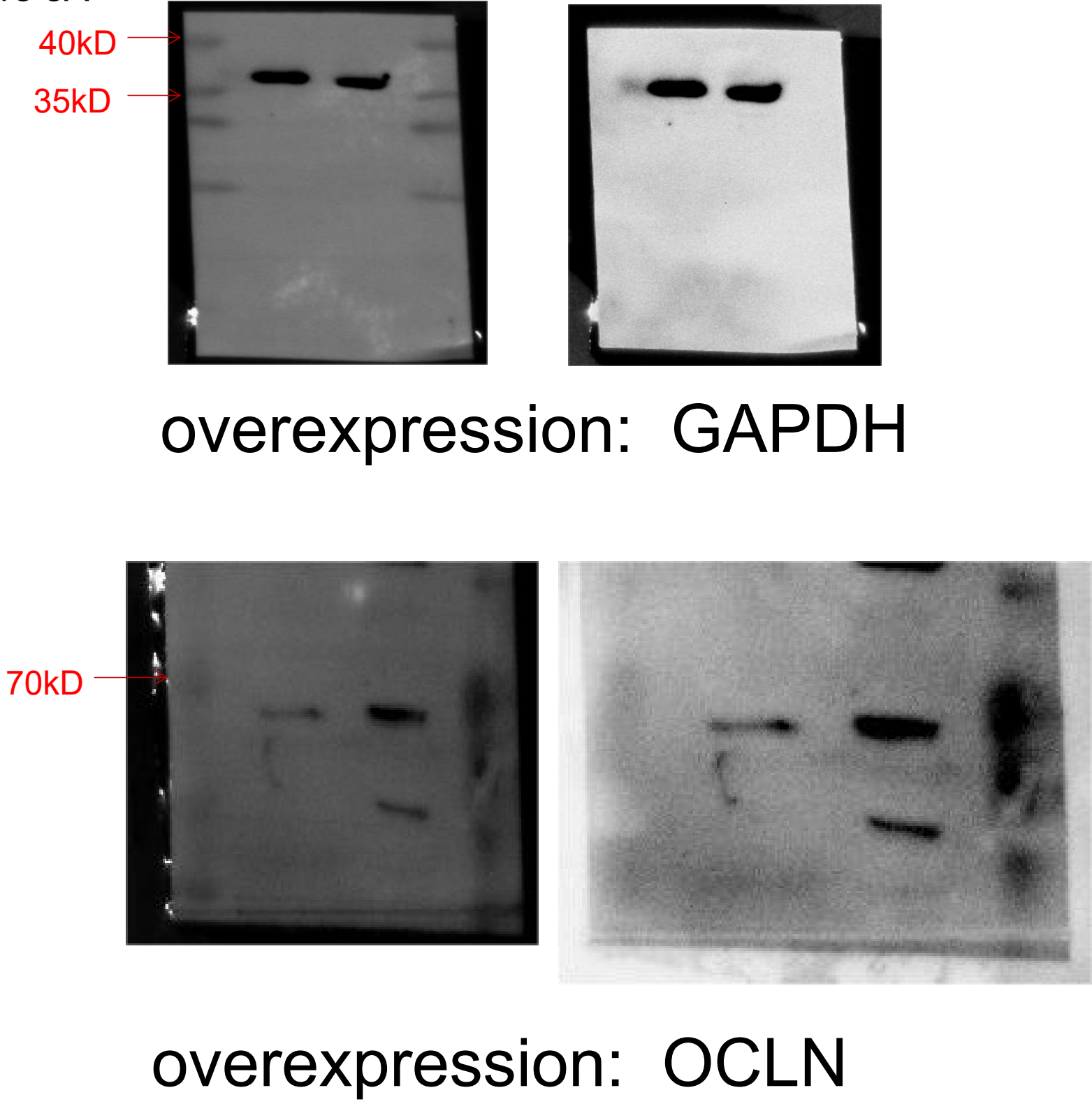

Supplement: Supplementary Figure S3 — Original image of blots/gels for Figures 7A , 8A . [file DataSheet_1.zip › Figure S3.pdf]

Figure 7C

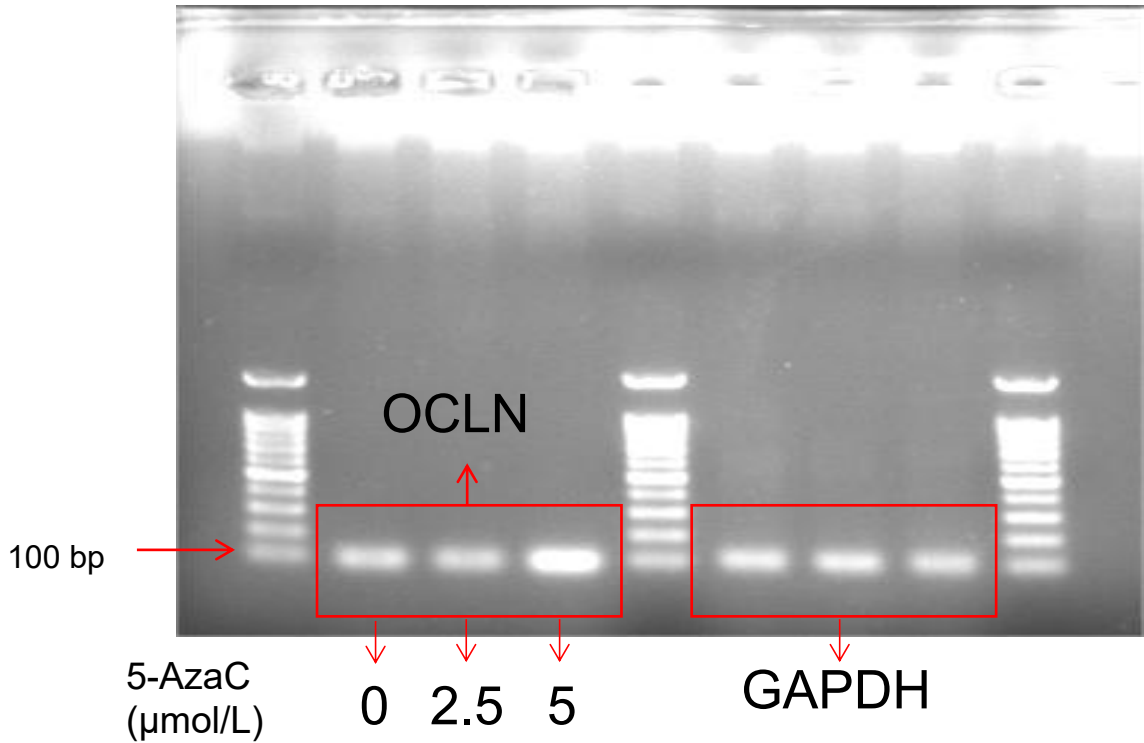

MSP

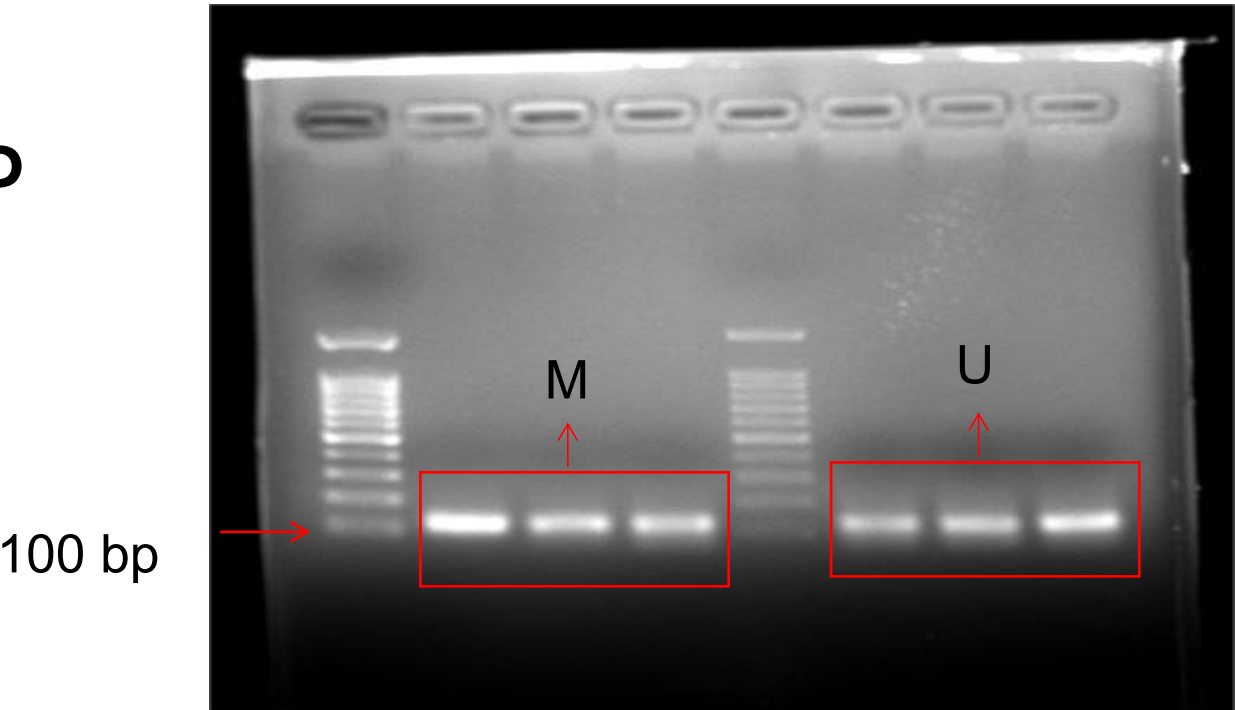

Figure 7D

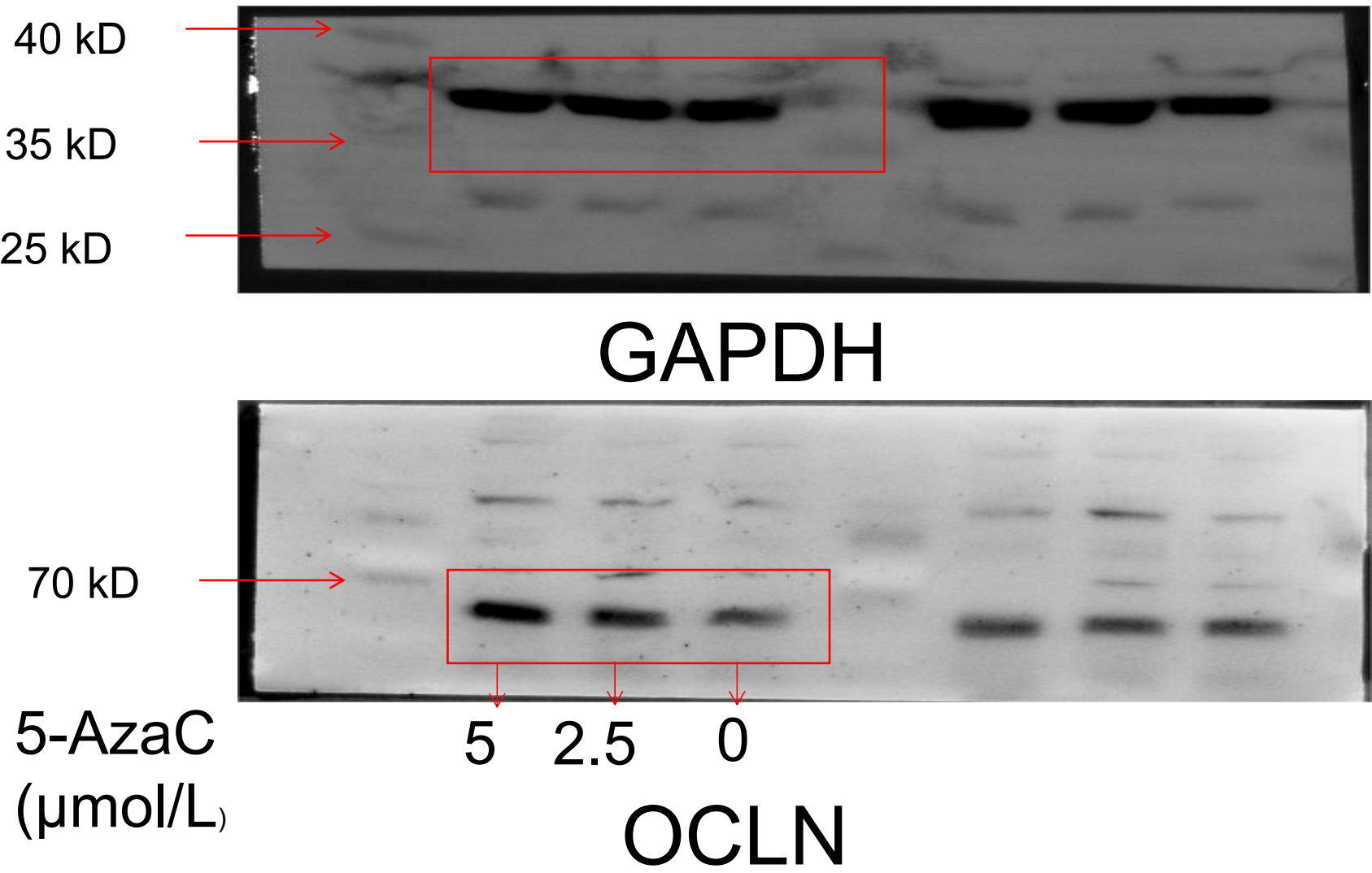

Supplement: Supplementary Figure S3 — Original image of blots/gels for Figures 7A , 8A . [file DataSheet_1.zip › Figure S4.pdf]
